# HyperArc radiotherapy for unresectable benign orbital tumours—cohort study and dosimetric comparison study

**DOI:** 10.1093/bjro/tzaf011

**Published:** 2025-05-10

**Authors:** Alasdair Innes Simpson, Caoimhe Henry, Ronan M Valentine, Richard Ferguson, Adam L Peters, Owen O’Brien, Sarah Al-Ani, Paul Cauchi, Stefano Schipani

**Affiliations:** Tennent Institute of Ophthalmology, Gartnavel General Hospital, Glasgow G12 0YN, United Kingdom; School of Cancer Sciences, University of Glasgow, Glasgow, G61 1QH, United Kingdom; Tennent Institute of Ophthalmology, Gartnavel General Hospital, Glasgow G12 0YN, United Kingdom; Beatson West of Scotland Cancer Centre, Glasgow G12 0YN, United Kingdom; Beatson West of Scotland Cancer Centre, Glasgow G12 0YN, United Kingdom; School of Cancer Sciences, University of Glasgow, Glasgow, G61 1QH, United Kingdom; Beatson West of Scotland Cancer Centre, Glasgow G12 0YN, United Kingdom; Tennent Institute of Ophthalmology, Gartnavel General Hospital, Glasgow G12 0YN, United Kingdom; Tennent Institute of Ophthalmology, Gartnavel General Hospital, Glasgow G12 0YN, United Kingdom; Tennent Institute of Ophthalmology, Gartnavel General Hospital, Glasgow G12 0YN, United Kingdom; School of Cancer Sciences, University of Glasgow, Glasgow, G61 1QH, United Kingdom; Beatson West of Scotland Cancer Centre, Glasgow G12 0YN, United Kingdom

**Keywords:** HyperArc, radiotherapy, orbital tumour, VMAT

## Abstract

**Objective:**

To report the clinical application, dosimetric features, efficacy, and toxicity profile of HyperArc (HA) for benign orbital tumours not amenable to surgical resection.

**Methods:**

A retrospective interventional cohort study. Gross target volume included the radiologically evident tumour and the optic nerve (excluded in case of haemangioma). Dosimetry was compared between HA and volumetric modulated arc therapy (VMAT) radiotherapy. Patients were treated with HA and followed-up clinically and radiologically for response and toxicity assessment.

**Results:**

Eight patients were included in our study, six patients with an optic nerve sheath meningioma, one cavernous haemangioma and one orbital schwannoma. All patients demonstrated tumour regression, mean tumour volume prior to treatment of was 4916 mm^3^ and reduced to 3239 mm^3^ (*P* = .03). Three of eight patients showed improvement of visual acuity, three retained excellent pre-treatment vision and two patients had a reduction of vision. HA and VMAT planning target volume coverage dosimetry was similar (D95%: 98.7% and 98.6%, *P* > .05). The dosimetry of the contralateral lens (32.2 vs 69.8 Gy), lacrimal gland (1.7 vs 7.8 Gy), optic nerve (9.0 vs 26.6 Gy), nasal cavity (10.2 vs 20.6 Gy) and ipsilateral temporal lobe (4.9 vs 11.6 Gy) was significantly improved (*P* < .001) with HA.

**Conclusion:**

This is the first reported clinical application of HA for benign orbital tumours. HA was an effective and well tolerated treatment modality. HA offered better dosimetry for some of the OARs compared to VMAT.

**Advances in knowledge:**

This is the first article reporting the use of the HA system for planning and delivery of radiotherapy for orbital tumours.

## Introduction

Tumours of the orbit, particularly those of the orbital apex are in close proximity to vital visual structures, such as the optic nerve and ophthalmic artery. Thus, even a small tumour can have a significant impact on visual function. Furthermore, the surgical resection of such tumours is challenging and carries significant risks. Radiotherapy has increasingly become the treatment modality of choice as an alternative or adjunctive to surgical resection. Advances in radiotherapy have led to more precise delivery systems that minimize irradiation of surrounding structures. Several forms of radiotherapy, such as fractionated stereotactic radiotherapy and stereotactic radiosurgery (single-session), have been described and may be delivered by an array of techniques.^1^

Volumetric Modulated Arc Therapy (VMAT) is one such technique that enables stereotactic radiotherapy to deliver radiation produced by a linear accelerator. It makes use of recent innovations, such as a multi-leaf collimator which allows for continual shaping of the radiation dose to the tumour volume. Varying the gantry speed and dose rate delivery further increases the modulation of therapy, aiding precision. HyperArc (HA, Varian Medical Systems, Palo Alto, CA) is an advanced planning and dose delivery technique originally used for the treatment of brain metastases.^1^ The conventional VMAT technique delivers radiotherapy doses with two coplanar arcs. HA has been specifically designed to deliver multiple coplanar and non-coplanar arcs, by altering the couch angle, thus further improving the target dose conformity and reducing collateral dose exposure to surrounding tissues. This technique has been principally used for the treatment of brain metastases and has been found to be as effective as conventional VMAT and Gamma Knife, and may be superior with regards to dosimetric parameters.^2–4^ The advantages of HA consist in the use of a non-invasive stereotactic immobilization system for the head, the high level of dose conformity and the rapid treatment time compared to other stereotactic techniques (eg, Gamma Knife). HA has been proposed for the treatment of conditions other than brain metastases, such as recurrent carcinoma of the nasopharynx.^5^

We aimed to evaluate the clinical outcomes and toxicity of HA for the treatment of unresectable benign orbital tumours and the dosimetric features in comparison to VMAT.

## Methods

Patients were referred to the ocular oncology department with suspected orbital masses. A clinical diagnosis of compressive optic neuropathy was made based on best corrected visual acuity, pupil reactions, visual field, Ishihara colour vision and optic disc appearance. All patients underwent contrast enhanced MRI of the head and orbit. A multidisciplinary team with radiation oncology, radiology, pathology, and ophthalmology specialists reviewed all the cases, made diagnosis and indicated radiotherapy as primary treatment.

**Figure 1.**
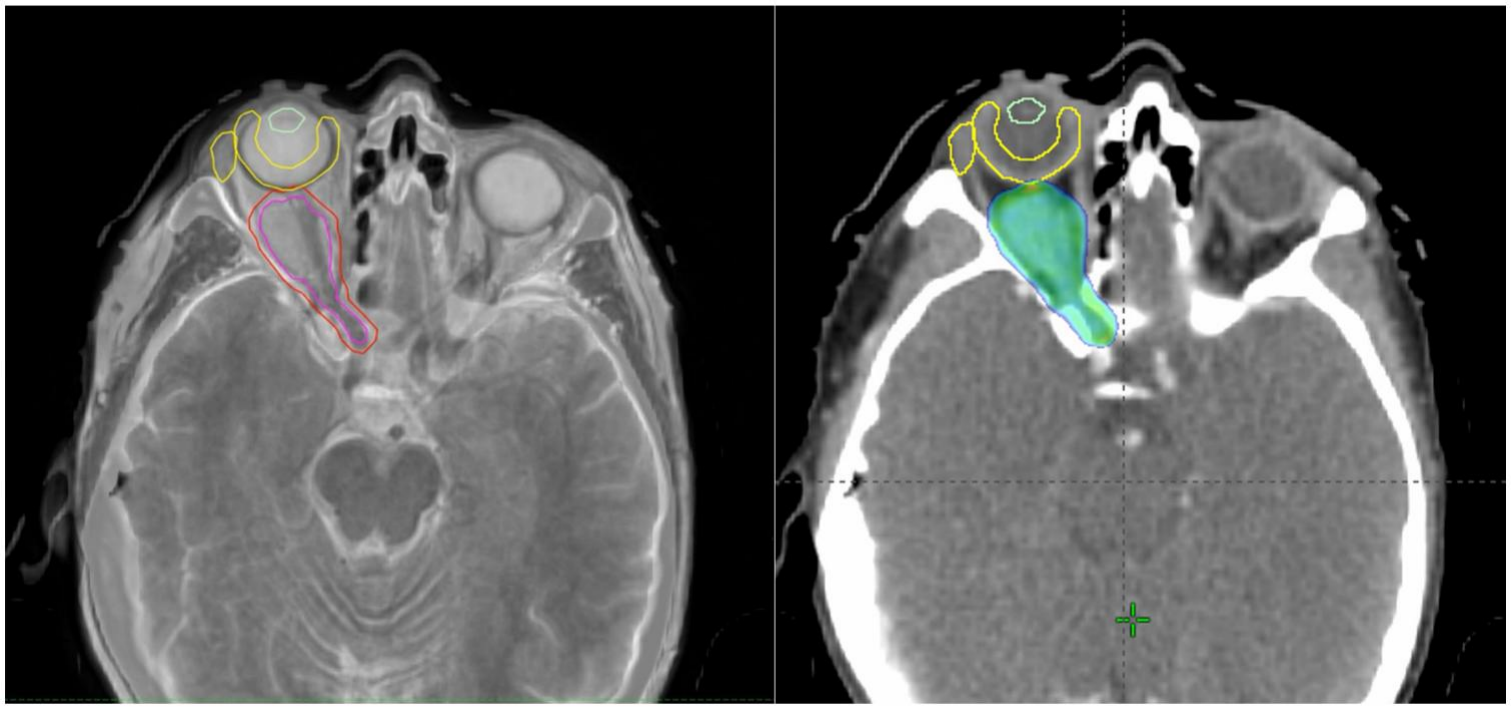
Patient (n.6) with right optic nerve sheath meningioma. Rigid fusion of simulation CT and diagnostic MRI. GTV (magenta) including the tumour and the optic nerve, PTV (red) and some of the OARs (lens, retina, lacrimal gland) are shown (left). The Hyperarc plan with 95% isodose (47.88 Gy) is shown (right).

Informed consent was obtained prior to initiating treatment. Patients were immobilized in a stereotactic non-invasive thermoplastic mask. A simulation contrast enhanced CT was acquired with 1 mm slices. Rigid registration of simulation CT and diagnostic MRI was used for radiotherapy planning purpose. Gross target volume (GTV) was defined as the radiologically evident tumour and the optic nerve, which was excluded in case of haemangioma. A 2 mm margin was added to the GTV to create the planning target volume (PTV). The following Organs at Risk (OARs) were contoured: brain, optic chiasm, pituitary, lachrymal gland, lens and retina. The HA technique with a maximum of four radiation beams consisting of one coplanar arc or semi-arc and three non-coplanar semi-arcs was used for treatment planning and delivery. A dose of 50.4 Gy in 28 fractions was prescribed to the PTV. The following dose constraints for the OARs were used for plan optimization: lachrymal gland Dmean 5 Gy, retina Dmean 10 Gy, lens Dmax 6 Gy, brain Dmean 5 Gy, pituitary Dmean 10 Gy, optic chiasm Dmean 10 Gy. An example of an HA radiotherapy plan is shown in Figure 1.

Separate VMAT plans for each patient, consisting of two coplanar arcs, were generated retrospectively by the radiotherapy physics department. PTV coverage and dose to OARs were generated for the HA and VMAT plans, allowing comparisons to be made and statistical significance tested via a student’s t-test and Wilcoxon rank sum test respectively. Statistical significance was indicated by a *P* < .05.

Patients were treated with HA using the TrueBeam^®^ linear accelerator (Varian Medical Systems, Palo Alto, CA) for 5 consecutive days per week and kV on board imaging was used for daily set-up verification. Patients were followed up clinically and radiologically for response and toxicity assessment.

## Results

Eight patients were treated with multisession HA between the years of 2018 and 2022. Table 1 details the patient’s characteristics. All eight patients were female; median age was 41 (27-59). All cases were unilateral (three right, five left orbits). Median duration of follow-up was 46 months with a range of 22-77. Patients presented with a range of symptoms, including headache, disturbed vision, diplopia and awareness of proptosis, with considerable variation in duration of symptoms prior to treatment varying from 1 to 24 months. None of our patients had any involvement of other cranial nerves of the orbit.

**Table 1.** Demographics and clinical characteristics at presentation to the orbital clinic.

Patient no.	Age at diagnosis	Gender	Laterality	Signs and symptoms	Duration of symptoms (months)	BCVA	Diagnosis
1	40	Female	Left	Headache and diplopia	6	6/6	ON sheath meningioma
2	38	Female	Left	Blurred vision and headache	24	HM	ON sheath meningioma
3	56	Female	Left	Blurred vision and headache	13	6/24	ON sheath meningioma
4	38	Female	Left	Headache and visual obscurations	1	6/4	ON sheath meningioma
5	27	Female	Right	Blurred vision	2	6/12	Cavernous haemangioma
6	42	Female	Left	Headache and proptosis	12	6/9	Schwannoma
7	56	Female	Right	Proptosis and blurred vision	24	6/9	ON sheath meningioma
8	59	Female	Right	Asymptomatic	6	6/12	ON sheath meningioma

Abbreviations: BCVA = best correct visual acuity (Snellen); HM = hand movements.

Orbital schwannoma was suspected in one case, based on an MRI scan. This enveloped the medial rectus and thus was felt not amenable to surgical resection without complete resection of the medial rectus. A biopsy was taken to confirm the diagnosis, as the tumour was relatively anterior in the orbit. This confirmed the diagnosis of orbital schwannoma. Diagnosis in the other seven cases was based on radiological features. Six patients were diagnosed with optic nerve sheath meningioma. One patient was diagnosed with cavernous haemangioma. Radiological diagnosis was based on the location, morphology and signal intensity in the MRI imaging studies. For optic nerve sheath meningioma; isointense to grey matter on T1 and T2-weighted images, avid homogeneous enhancement which contrasts with the non-enhancing optic nerve, resulting in “tram-track sign” on axials. For cavernous haemangioma, a well-defined rounded or oval morphology of intraconal lesion isointense to muscle on T1 and hyperintense on T2-weighted images, with enhancement, suggests a slow flow venous malformation/cavernous haemangioma. For the cavernous haemangioma, the tumour was located at the orbital apex and therefore was not amenable to surgical resection.

### Changes in tumour volume after treatment

**Figure 2.**
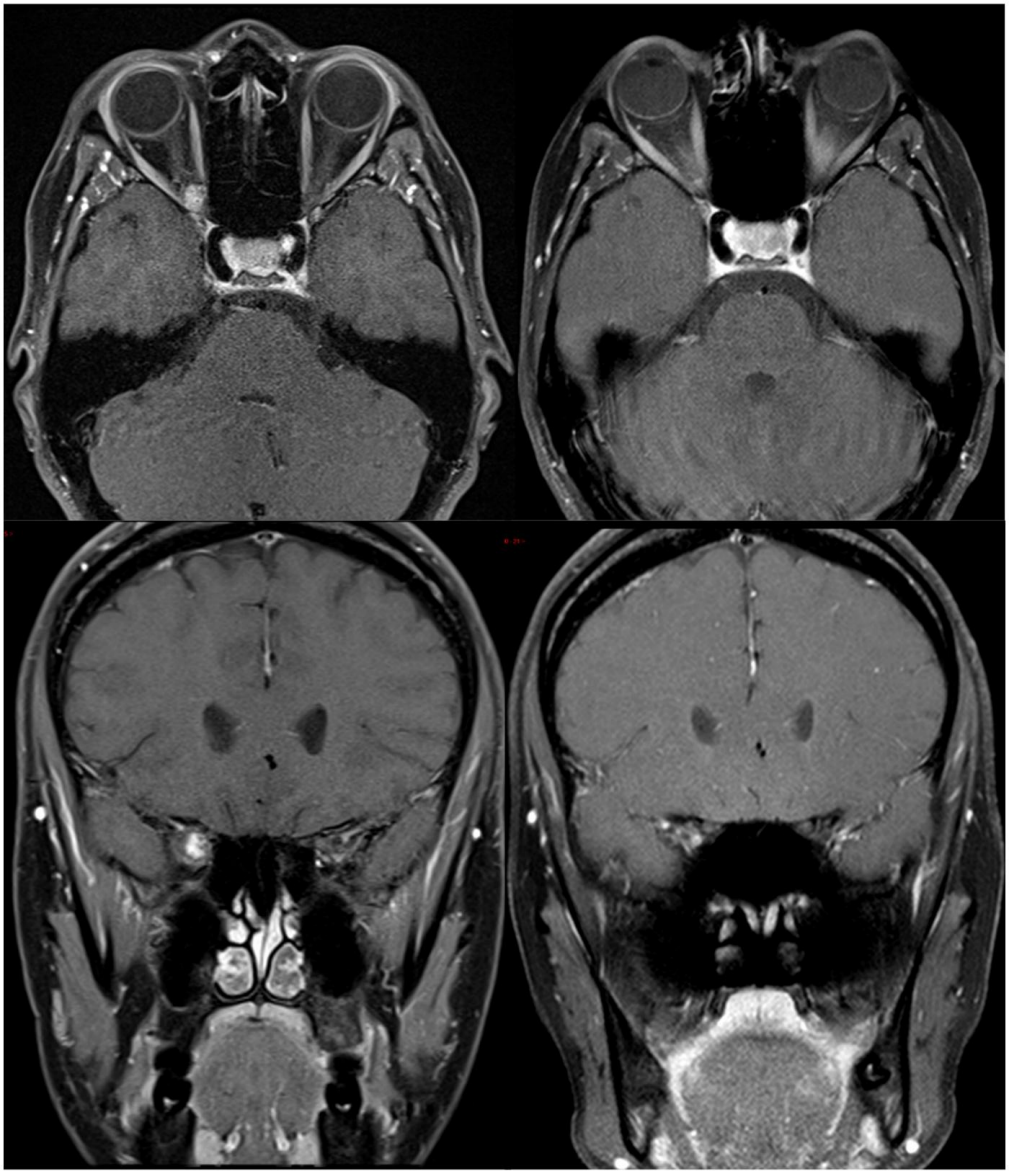
Patient 5. Twenty-seven-years-old female experiencing blurred vision. (Top left) Pre-treatment axial T1 weighted fat-suppressed post-contrast MR image showing a well-defined, homogeneously enhancing intraconal mass at the right orbital apex. (Top right) Post-radiotherapy axial T1 weighted fat-suppressed post-contrast MR. (Bottom left) Pre-treatment coronal T1 weighted fat-suppressed post-contrast MR. (Bottom right) Post-radiotherapy MR demonstrates reduction in size of the intraconal right orbital apex mass.

**Figure 3.**
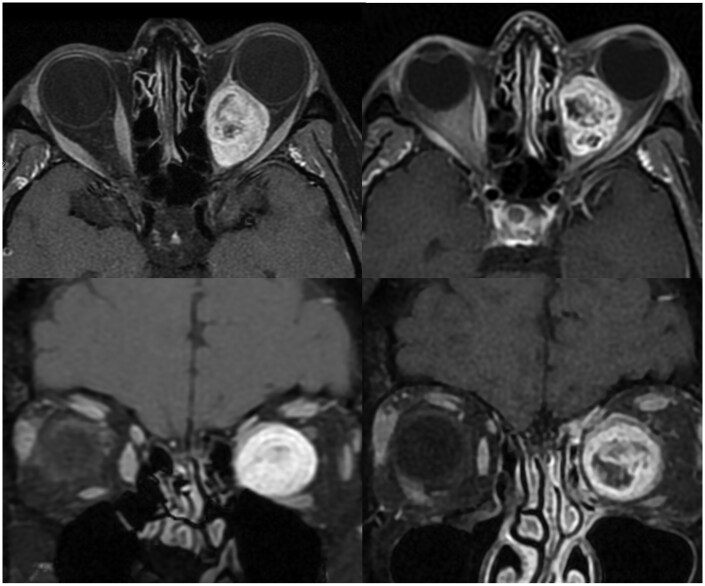
Patient 6. Forty-two-years-old female presented with proptosis and headache. Biopsy confirmed an orbital schwannoma. (Top left) Axial T1 weighted fat supressed post contrast MR demonstrated a left conal/intraconal mass. (Top right) Post radiation axial image demonstrates increasing enhancement of solid components and reduction in non-enhancing cystic components. (Bottom left) Coronal MR with lesion causing marked displacement of the optic nerve. (Bottom right) Coronal scan post-radiation.

The volumes of the lesions prior to and following treatment are shown in Table 2. The tumour volume pre-treatment ranged from 492 to 6554 mm^3^. All patients experienced a reduction in tumour volume, as confirmed by MRI at latest follow-up (see Figures 2 and 3 for illustrative results). The median change in tumour volume was 944 mm^3^ (456 to 1786 mm^3^) or −29% (−15% to −95%). Median tumour volume prior to treatment was 4115 mm^3^ and reduced to 3239 mm^3^ (*P*-value = .03).

**Table 2.** Clinical characteristics of patients before and after radiotherapy.

Patient no.	BCVA		Proptosis (mm)		Disc appearance		RAPD		Tumour volume (mm^3^)			Follow-up (months)
	Before	After	Before	After	Before	After			Before	After	Change	
1	6/6	6/6	6	6	Swollen	Normal	−	−	6553.9	4768.3	−27%	77
2	HM	HM	0	0	Pale	Pale	+	±	1675.5	1420.3	−15%	67
3	6/24	6/30	3	3	Swollen	Pale	+	±	1661.1	1304	−21%	53
4	6/4	6/4	1	0	Swollen	Normal	−	−	1628.4	1118.3	−31%	44
5	6/12	6/6	0	0	Temporal pallor	Temporal pallor	+	−	491.8	26.8	−95%	48
6	6/9	6/9.5	5	0	Swollen	Normal	+	+	10176	4874.1	−52%	42
7	6/9	6/19	3	4	Pale	Pale	+	+	7261.1	4665.2	−36%	32
8	6/12	6/6	5	4	Swollen	Normal	−	−	9876.2	7735.7	−22%	22

Abbreviations: BCVA = best correct visual acuity (Snellen); RAPD = relative afferent pupillary defect; + = easily elicited; ± = barely detectable; − = absent.

### Ophthalmic outcomes

Three patients showed an improvement in visual acuity, three retained excellent pre-treatment acuity and two had a drop in acuity (Table 2). The patients were assessed in different hospitals, resulting in inconsistency in the type of visual fields used. Four patients had reduced colour vision on presentation; this improved following treatment in one patient. Proptosis was recorded in six patients prior to radiotherapy, three having a recorded reduction, two unchanged and two worse. Relative afferent pupillary defect was detected in five patients prior to radiotherapy. In one case this totally resolved following treatment.

The five patients with a reduction in tumour volume had stabilization or a small increase in visual acuity with no further intervention planned (Figures 1-3). The patient with a schwannoma had an initial tumour volume (10 176 mm^3^) and slow clinical improvement with initial worsening visual acuity and increased proptosis. However, at the most recent review (42 months following completion of radiotherapy), proptosis had resolved, colour vision and visual acuity were at pre-treatment baseline.

### Radiation dosimetry comparison HA vs VMAT

**Figure 4.**
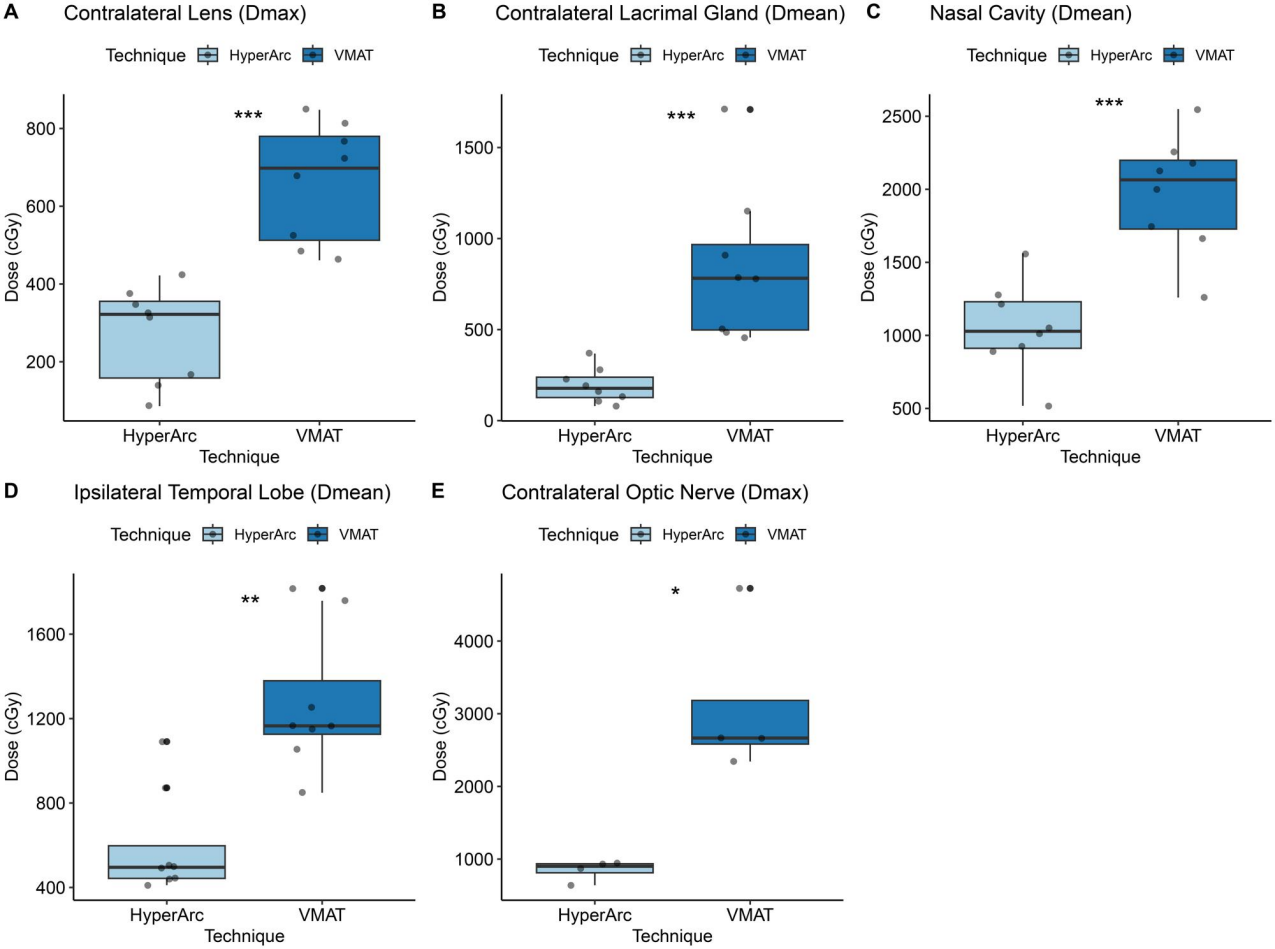
Box plot of organ at risk results comparing of VMAT and HyperArc dosimetry.

Overall the HA plans provided good dose coverage of the PTV and met the constraints for the OARs. There was no statistical difference in PTV coverage between the HA and VMAT plans (Table 3). Doses to critical OAR structures such as the contralateral lens, contralateral lacrimal gland, contralateral optic nerve, ipsilateral temporal lobe and nasal cavity were significantly lower with HA compared to VMAT (*P-*value < .001) (Figure 4). All other ipsilateral structures and other unpaired OAR structures showed no difference with regard to dosimetry (Table 4).

**Table 3.** Comparison of planning target volume using HyperArc vs VMAT.

PTV structure	HyperArc (median %)	VMAT (median %)	*P*-value^a^
D99%	97.5	97.35	.809
D95%	98.75	98.6	.778
D50%	100.85	100.05	.409
D5%	103.05	102.65	.603
D2%	103.8	103.1	.599

a Student *t*-test.

**Table 4.** Comparison of organ at risk dosimetry for HyperArc vs VMAT.

OAR structure	*N*	HyperArc (cGy), *N* = 8^a^	VMAT (cGy), *N* = 8^a^	***P*-value** ^b^
Contralateral Lens (Dmax)	16	322 (158-356)	698 (513-780)	**<.001**
Ipsilateral Lens (Dmax)	16	457 (370-547)	506 (445-557)	.67
Contralateral Lacrimal Gland (Dmean)	16	178 (127-239)	782 (498-967)	**<.001**
Ipsilateral Lacrimal Gland (Dmean)	16	433 (340-494)	458 (418-501)	.38
Ipsilateral Macula (Dmax)	14	2725 (1662-2887)	2503 (1764-2865)	>.99
Ipsilateral Retina (Dmean)	16	1011 (930-1222)	1044 (921-1132)	.96
Ipsilateral Optic Disc (Dmax)	12	3484 (2334-4400)	2990 (2389-4253)	.82
Optic Chiasm (Dmax)	16	3561 (14734358)	3831 (1462-4732)	.80
Optic Chiasm (Dmean)	16	1099 (432-1280)	1169 (563-1483)	.51
Contralateral Optic Nerve (Dmax)	8	901 (811.8-935.3)	2666 (2583-3183)	**<.05**
Ipsilateral Optic Nerve (Dmax)	8	4245.5 (2826.8-4952.3)	4293.5 (2988.8-4850.8)	.99
Brain (Dmean)	16	224 (213-231)	189 (177-230)	.33
Brainstem (Dmean)	16	211 (190-247)	201 (178-232)	.88
Pituitary Gland (Dmax)	16	2367 (1415-2794)	2487 (1943-2874)	.88
Nasal Cavity (Dmean)	16	1028 (912-1230)	2065 (1728-2199)	**<.001**
Ipsilateral Temporal Lobe (Dmean)	16	496 (443-598)	1166 (1126-1379)	**.001**

a Median (IQR); *n* (%).

b Wilcoxon rank sum test.

### Toxicity

Following radiotherapy one patient reported no side effects, the other seven patients reported mild G1 level side effects including eye pain, photophobia or dry eyes. No late ocular complications were reported in any subject. In particular, no cases of radiation induced retinopathy or optic neuropathy were recorded.

## Discussion

Currently, the active treatment options for benign orbital tumours include surgical resection, external beam radiotherapy, brachytherapy or a combination. Surgical microdissection can allow acute decompression of the optic nerve; however, operating within the orbital apex is challenging due to the small confines of space and close anatomical relations of vital structures.^6^ Thus surgical intervention is associated with significant risk of blindness.^7^^,^^8^ Various surgical approaches have been described and these can be broadly categorized into superior, lateral, medial, inferior, endoscopic endolaser and transorbital neuroendoscopic approaches.^9^ An alternative approach to direct resection has been bony decompression of the orbit, however there remains debate as to the effectiveness of these procedures to adequately relieve compression of the optic nerve.^10^^,^^11^ Given the surgical challenges ionizing radiotherapy is a vital alternative for non-resectable and inaccessible tumours.

Various types of radiotherapy for both malignant and benign orbital tumours exist and can be broadly categorized into internal radiotherapy, that is, brachytherapy and external beam radiotherapy, which typically includes techniques such as intensity-modulated radiation therapy and volumetric modulated arc therapy (VMAT).^12^ The goal of all radiotherapy is to deliver maximal irradiation to the tumour and minimize exposure to healthy tissue, often termed OARs. Normal ocular structures are variably radiation sensitive, with the crystalline lens being particularly sensitive and orbital bone and sclera relatively resistant.^12^ All forms of radiotherapy pose a potential risk to the optic nerve. Given the close proximity of tumours to the optic nerve it has been shown in stereotactic radiotherapy to receive equal doses of irradiation to that of the tumour and studies suggest the optic nerve may be more sensitive to irradiation damage than other cranial nerves.^13^^,^^14^ Various studies have sought to identify a threshold as to when radiation optic neuropathy will occur; however, multiple factors appear relevant including total dose, dose size, dose frequency and degree of optic nerve compromise caused by tumour compression and as such no clear threshold has been established.^12^ There remains limited experience of radiotherapy delivery to well circumscribed benign tumours of the orbital apex, meaning that experience is often inferred from the neurosurgical literature.

Fractionated Stereotactic Radiotherapy (SRT) generally uses low dose per fraction delivered over multiple sessions. SRT is a form of external beam radiotherapy with many delivery techniques now available including those using photons (gamma rays or X-ray) or particles (protons or neutrons). Linear accelerators being used to generate high energy X-rays are the most common type, and this underpins the HA technique. SRT provides an alternative to surgical resection for many benign and malignant tumours of the orbit.^12^^,^^15^ Though the orbit and particularly the orbital apex contain many visually vital structures which can be damaged by irradiation, hence the ever more precise nature of radiotherapy delivery means that this is often considered safer than surgical resection.^15^ An alternative to SRT is Stereotactic Radiosurgery (SRS) which can be delivered in a single or relatively few ultra-high dose fractions in tightly conformed target volumes. Single session delivery may increase the risk of optic nerve damage as it has been observed that irradiation with more than 8 Gy in a single session can lead to radiation optic neuropathy.^13^^,^^16^ So, a compromise has been found in the use of multisession radiosurgery using modalities such as CyberKnife and Gamma Knife (Accuray, Inc., Sunnyvale, CA), usually delivered in 3 fractions.^17–19^

The HA technique is a relatively new SRT delivery system. HA offers superior efficiency compared to conventional Linac-based VMAT techniques without compromising safety.^20^ Ohira et al considered the performance of HA in the treatment of multiple or single brain metastasis and found it to be superior in terms of conformity and dose fall-off compared to conventional VMAT.^1^ This finding was supported by Vergakasaiva et al, who also found HA to be superior to manual VMAT planning and comparable to Gamma Knife. Synder et al have proposed the use of HA for skull base meningiomas in close proximity to the optic nerve rendering them unresectable, finding it to be beneficial both in terms of efficacy and brain tissue sparing.^21^ Beyond the use in intracranial malignancy it has also been proposed for the use of nasopharyngeal carcinomas and out performs RapidArc.^5^ However, to our best knowledge, no study has reported the use of HA in the treatment of orbital benign tumours. Gamma Knife and CyberKnife are not available in our institution, so we decided to compare the dosimetry of HA and VMAT. Our data show that HA and VMAT offer similar target volume coverage. HA significantly improves the dosimetry of the contralateral organs, the nasal cavity and the ipsilateral temporal lobe. This dosimetric advantage might not be relevant in terms of early toxicity but could be helpful to mitigate the long-term risk of radiation induced cancer associated with the low-dose spillage in such patients who are generally young and treated for a benign condition.

In this series all patients experienced an improvement in their symptoms and tumour regression. In these six cases, proptosis stabilized or reduced. There have been no instances of long-term complications and any complications that did occur during the treatment regime such as orbital pain, dry or watery eyes completely resolved. However, in the patient with orbital schwannoma the tumour size increased by 3% at 2 years with worsening of visual acuity and an increase in proptosis. In this instance the tumour actually increased by 54% at 12 months, before involuting. At most recent review, 42 months following completion of HA treatment, the proptosis had completely resolved and vision improved to baseline. This was by far the largest of all the tumours treated in this series. This behaviour of schwannomas has been observed by Young et al who comment that their experience with treating Schwannomas with multisession Gamma Knife is that of an initial increase in tumour size followed by a slow decrease to its original volume.^19^ Kim et al reported differing response dependent on tumour type, with cavernous haemangiomas being particularly responsive with reduction in volume by over 70%.^18^ With regard to the six optic nerve meningiomas treated there appeared to be a trend that the bigger the tumour the greater the volume shrinkage. In our series the same total treatment dose was applied to every tumour. As evidence and experience grows it may be that total radiation dose can be tailored to tumour type and size, and selection of amenable tumours refined.

For orbital meningiomas a total treatment dose of 54 Gy in 33 fractions has been suggested by Finger et al with an expectation of poor long term visual prognosis.^12^ We observed that four of six patients with optic nerve sheath meningiomas had improved or stable visual acuity, one patient had a slight drop in acuity from 6/24 to 6/30 and the other patients vision dropped from 6/9 to 6/19. This finding challenges the view that the optic nerve is particularly radiosensitive. Although when consenting patients, a guarded prognosis for visual outcome, is recommended, a more optimistic outcome might cautiously be expected.

The main advantages of HA are the use of a stereotactic non-invasive immobilization mask, the high dose conformity with good sparing of the OARs and the potential rapid treatment time due to the high dose rate of the LINAC. When compared to other treatment modalities such as Gamma Knife or CyberKnife, the duration of sessions is markedly reduced. In our centre each arc can be delivered in under a minute and HA sessions take 8-12 min in total. During SRT techniques such as multi-sessional Gamma Knife patients wear a skull fixated headframe while an inpatient for three days and this is acknowledged to be a potential drawback.^19^^,^^22^ In contrast, non-invasive personalized moulded face masks are created and worn for immobilization during out-patient HA treatment sessions.

There are limitations to our study as it is of course a small series, treating a relatively heterogeneous group of patients. There is also a difference in length of follow-up, though all the patients had at least 22 months follow-up. Only one of our patients had their diagnosis verified with a biopsy and histopathology. In the other cases, diagnosis was made on radiological findings. This is not unusual in comparable studies as tumours at this location are challenging and risky to biopsy. We acknowledge that dynamic MRI scans may have aided diagnosis but this is not our routine practice, and as a retrospective study represents a real world approach to such tumours. Another limitation is the lack of comparable visual fields: two of the eight patients had visual acuities unlikely to be compatible with reproducible visual fields in any case. The authors have made arrangements that all future patients will undergo formal Goldmann visual fields prior to radiotherapy even if this means referring to another hospital.

## Conclusions

This study has suggested that the delivery of 50.4 Gy in 28 fractions using the HA planning and delivery technique is a feasible and effective treatment for non-resectable benign orbital tumours. HA offered better dosimetry for some of the OARs compared to VMAT. The toxicity profile was acceptable with only mild and short-lived side effects reported. All patients with evidence of optic nerve compression experienced tumour shrinkage, and relief of symptoms and the majority were found to have improvement or stabilization of visual acuity. This treatment modality avoids the risks associated with surgical resection. Future studies are required to validate these findings in a larger cohort of patients.

## Funding

No specific funding was used to undertake this research.

## Conflicts of interest

The authors declare that they have no conflict of interest.
